# The role of adipokines in connective tissue diseases

**DOI:** 10.1007/s00394-012-0370-0

**Published:** 2012-05-15

**Authors:** Robert Krysiak, Gabriela Handzlik-Orlik, Boguslaw Okopien

**Affiliations:** Department of Internal Medicine and Clinical Pharmacology, Medical University of Silesia, Medyków 18, 40-752 Katowice, Poland

**Keywords:** Adipose tissue, Adiponectin, Leptin, Resistin, Rheumatoid arthritis, Systemic lupus erythematosus

## Abstract

**Objective:**

To discuss the relationship between adipokines and connective tissue diseases, by putting special emphasis on the potential role of leptin, adiponectin, resistin, and other adipose tissue products in the pathogenesis of rheumatoid arthritis and systemic lupus erythematosus and on possible application of adipokine-targeted therapy in the treatment of these disorders with emphasis on the recent findings.

**Methods:**

PubMed literature search complemented by review of bibliographies listed in identified articles.

**Results:**

Most of the data presented by different research groups showed changed levels of leptin, adiponectin, and resistin and occasionally also other adpokines in rheumatoid arthritis and systemic lupus erythematosus. The relationship between the remaining connective tissue diseases and adipokines is less documented.

**Conclusions:**

Plasma levels of adipokines might tell us too little about their role in connective tissue disorders, whereas adipokine effects on synovial tissues might differ from their known metabolic or cardiovascular effects, which implies that some re-appraisal of adipokines role may need to take place. It still remains obscure whether the observed disturbances in various adipokine systems in subjects with connective tissue diseases contribute to their development or only reflect the presence or activity of inflammatory process, which itself is induced by other pro-inflammatory factors.

## Introduction

Adipokines, proteins produced by the white adipose tissue (WAT), have attracted the attention of scientists of various specialties since 1994, when the first adipokine—leptin was discovered. For decades, adipose tissue had been regarded only as a storage depot for body energy, mechanical defense against injuries, and a thermoregulator [1]. The discovery of leptin, and a year later of adiponectin, markedly broadened our knowledge on the contribution of adipose tissue to whole-body homeostasis. Although, initially, adipokines were considered to determine their association with eating disorders and diabetes, later studies revealed that adipose tissue products play an important role in the regulation of immune response and systemic inflammatory processes. Taking into consideration their action on inflammation, these peptides may be divided into those inducing mainly pro-inflammatory [leptin, resistin, interleukin (IL)-6, tumor necrosis factor α (TNF-α)] or predominantly anti-inflammatory (adiponectin, IL-1 receptor antagonist, IL-10) effects [2].

The involvement of adipokines in the regulation of inflammatory processes, in light of still obscure pathogenesis of connective tissue diseases and insufficient control of these disorders by generally accepted medications, aroused the interest of scientists in a possible causative role of abnormal adipokine production in the pathogenesis of autoimmune disorders and as potential targets for treatment of these disorders. It was found that articular and extra-articular changes associated with rheumatoid arthritis (RA) and reduced physical activity lead to the alteration in body fat distribution, which include “cachectic obesity”, a state where muscle loss is accompanied by fat gain, so that in early stages, body weight can remain stable [3]. Interestingly, underweight or normal weight have been associated with more active disease, while obesity has been accompanied by significantly less joint damage, both before and during the treatment [4]. Also systemic lupus erythematosus (SLE) pathogenesis may be related to body mass index (BMI) status, which is confirmed by the fact that two-thirds of patients suffering from this disease are overweight or obese, either before or after diagnosis [5].

### Physiological role of adipokines

#### Leptin

**Fig. 1:**
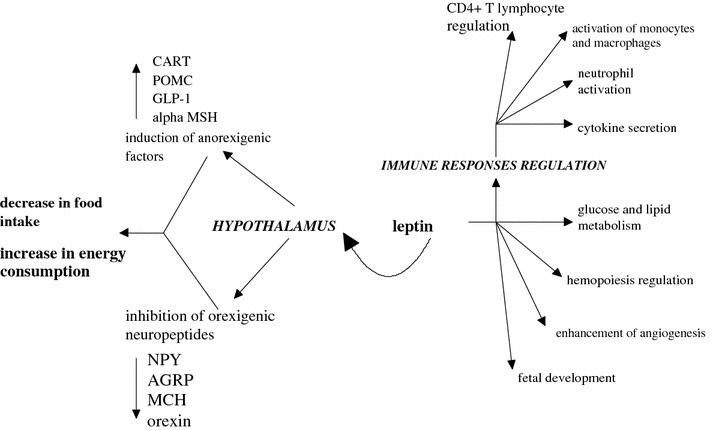
Model of leptin action on hypothalamus and immune response regulation (*CART* cocaine- and amphetamine-regulated transcript, *POMC* proopimelanocortin, *GLP*-*1* glucagon-like peptide-1, *MSH* melanocyte-stimulating hormone, *NPY* neuropeptide Y, *AGRP* agouti-related peptide, *MCH* melanin-concentrating hormone

Leptin is a 16 kDa non-glycosylated polypeptide hormone produced mainly by WAT, discovered by positional cloning of a single gene mutation in the *ob/ob* mouse. In humans, it is encoded by the *Lep* gene (an equivalent of the *ob* gene in mouse) [6]. Circulating levels of leptin correlate with BMI and the content of fat mass [7]. After crossing the blood–brain barrier, leptin reaches the hypothalamus, where it acts as a crucial regulator of feeding. Leptin is mainly regarded as a “starvation-hormone” signaling from the adipose tissue (AT) to the brain, indicating the size of the AT-stores [8] and it exerts its action by inducing the expression of anorexigenic factors and reducing the hypothalamic production of orexigenic peptides. However, leptin has been shown to influence a wide spectrum of other biological functions [7–14] (Fig. 1). Its production depends on the insulin levels, energy status, sex hormones, and a wide range of inflammatory mediators, including IL-1, TNFα, and leukemia inhibitory factor (LIF) [12, 15]. The fact that leptin synthesis and release is inhibited by testosterone and stimulated by ovarian sex steroids explains higher plasma leptin levels in women than in men, even after adjustment for BMI [16].

Numerous studies showed increased plasma leptin levels during infection and inflammation [17, 18], which is probably secondary to the enhanced production of IL-1β, TNFα, and IL-6. These data suggest that leptin plays a relevant role in immunity as it affects the function of immune cells by regulating cytokine production and polarizing T helper cells toward Th1 [18].

#### Adiponectin

Adiponectin is a 244-amino acid protein, produced mainly by WAT. The monomeric form (30 kDa) of adiponectin seems to occur only in the adipocyte, whereas oligomeric complexes circulate in plasma as low molecular weight trimers (LMW), middle molecular weight hexamers (MMW), and high molecular weight multimers (HMW) [19]. There is also a globular form of adiponectin, which arises from the cleavage of full-length adiponectin by leukocyte elastase [20]. Plasma adiponectin levels far exceed plasma concentrations of other AT products [19].

A physiological significance of adiponectin has not yet been fully explained. In opposition to most other adipocyte-derived cytokines (leptin, resistin, adipsin, etc.), adiponectin levels are decreased in obesity (probably through TNFα and IL-6 downregulation). On the other hand, increased adiponectin levels are observed in patients with anorexia nervosa and in fasted healthy subjects [21]. It is generally accepted that high adiponectin levels enhance insulin sensitivity, while low adiponectin plasma levels are associated with type 2 diabetes mellitus, dyslipidemia, and hypertension [22]. Thus, there is a negative correlation between low adiponectin levels and severity of the metabolic syndrome [23, 24].

**Fig. 2:**
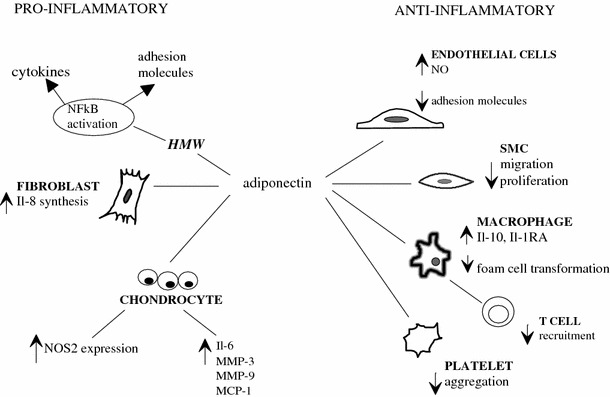
Bidirectional properties of adiponectin

In the majority of the studies conducted to date, adiponectin produced anti-inflammatory effects (Fig. 2). Interestingly, adiponectin inhibited TNFα and IL-6 production, while both TNFα and IL-6 suppressed adipocyte adiponectin production [2, 24], which suggests the existence of a negative feedback between adiponectin and pro-inflammatory cytokines. TNFα- and IL-6-suppressing and the remaining anti-inflammatory effects of adiponectin shown in Fig. 2 [12, 25–27] may contribute to the protection against CVD, which is lacking in obese subjects.

However, some reports surprisingly suggest pro-inflammatory action of adiponectin [28, 29] (Fig. 2). It has been evidenced that the serum concentration of adiponectin is elevated in patients with classic chronic/autoimmune inflammatory conditions, such as inflammatory bowel disease or type 1 diabetes mellitus [24]. Although elevated adiponectin levels could be explained by a compensatory response of organism against inflammation, some researchers find this explanation unconvincing and point to the role of adiponectin in NFκB activation [28, 29].

This bidirectional, anti- and pro-inflammatory effects of adiponectin may in part result from the changes in the relative proportion of its various isoforms. LMW adiponectin has been shown to block endotoxin-induced secretion of IL-6 and to induce IL-10 production, while MMW and HMW adiponectin has been found to stimulate monocyte chemoattractant protein-1(MCP-1) and IL-8 synthesis [30]. Interestingly, HMW adiponectin has been shown to serve not only as a predictor of future cardiovascular events in patients with coronary artery disease, but also as a marker for severity of CAD [31], whereas the MMW/HMW ratio, but not LMW or total adiponectin levels, correlated with the incidence of myocardial infarction [32]. These findings imply that measurement of adiponectin multimers adds significant value in assessing cardiovascular risk compared to total adiponectin alone and that the ratio of the isoforms may determine adiponectin action.

Certainly, it cannot be excluded that adiponectin production and secretion is regulated in a disease-dependent manner and that adiponectin action depends on a type of inflammatory disorder a patient suffers from.

#### Resistin

**Fig. 3:**
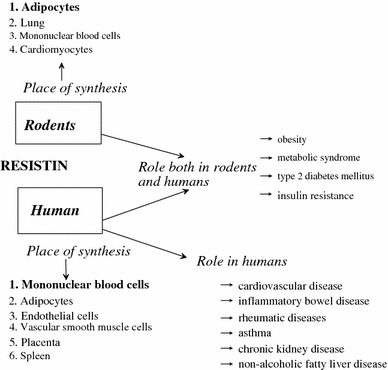
Synthesis and function of resistin in humans and rodents

Resistin is a 12.5-kDa member of cysteine-rich proteins called “resistin-like molecules” or “found in inflammatory zone” (FIZZ). It was initially described as an adipocyte-derived protein and a mediator of hepatic insulin resistance (IR) [33]. Interestingly, there are marked interspecies differences in the source of production and structure of this protein (Fig. 3). In mice, resistin is synthetized mainly by WAT [34]. In humans, on the other hand, WAT produces only small amounts of this protein [35], while relatively high levels of resistin mRNA levels are detectable in circulating mononuclear cells [36]. Physiological function of resistin may in part differ between humans and rodents [37], because of the fact that mouse and human resistin share only about 64 % sequence homology at the mRNA level and only 59 % identity at the amino acid structure [38]. In humans, the best documented is its role in regulating metabolic processes, adipogenesis, and inflammatory reactions. Some [39, 40], but not other [41, 42], authors found that plasma resistin levels positively correlate with obesity and other constituents of the metabolic syndrome. Moreover, high plasma resistin levels were found to correlate with impaired renal function in patients with chronic kidney disease [43], with the severity of inflammation in inflammatory bowel disease [44], and with increased risk for cardiac events in patients with congestive heart failure [45] (Fig. 3).

The presence of FIZZ molecules within or near inflammatory areas suggests the involvement of resistin in the development of inflammatory responses. In humans, resistin has a potency to induce the production of IL-6, IL-1β, and TNFα by peripheral blood mononuclear cells (PBMCs) [36, 46]. Also, synovial fluid cells express IL-6 and TNFα when stimulated by resistin. Similarly, pro-inflammatory cytokines increase resistin expression in PBMCs [47]. Bokarewa et al. [34] have demonstrated that the pro-inflammatory effects of resistin are mediated through the NF-κB signaling pathway.

#### Other adipokines

Recent years have witnessed the discovery of novel AT-derived peptides.

Visfatin, an adipokine produced mainly by visceral WAT, binds and activates the insulin receptor exerting its insulin-mimetic effect. The structure of visfatin is identical to pre-B-cell colony-enhancing factor (PBEF), the production of which is increased in animal models of acute lung injury and in neutrophils of septic patients [12, 21]. It is also synthesized in endotoxin-stimulated neutrophils, preventing apoptosis of these cells.

Vaspin is an adipose tissue–derived member of the serine protease inhibitor family with insulin-sensitizing effects, which seems to be a compensatory mediator for abrogating obesity and its inflammatory complications [48].

Omentin, a peptide highly and selectively expressed in visceral AT, is involved in the regulation of insulin action [49]. Isolated from the omentum of patients with Crohn’s disease, omentin is suggested to be implicated in the pathogenesis of chronic inflammatory diseases [48].

Chemerin is a chemoattractant protein almost exclusively expressed in adipose tissue and to much lesser extent by immune-competent cells. It is implicated in metabolic and immune homeostasis [50]. Its secretion in murine adipocytes has recently been reported to be induced by IL-1β [51].

Another novel adipokine, lipocalin 2 (LCN 2), is a glycoprotein principally produced by WAT, but also isolated from granules of neutrophils [52]. LCN2 induces apoptosis in hematopoietic cells, modulates inflammation and metabolic homeostasis [53]. It has recently been identified in chondrocytes, where TNFα is a main regulator of its expression.

Serum amyloid A3 (SAA3) belongs to the family of acute-phase serum amyloid A proteins. In humans, SAA3 is a pseudogene and its functional protein is unknown. In experimental studies, secretion of this adipokine is modulated by IL-1β in adipocytes and chondrocytes [54], while SAA3 induces transcription of matrix metalloproteinase-13 (MMP-13) [53].

## Results

### Rheumatoid arthritis

Rheumatoid arthritis (RA) is a chronic systemic inflammatory disease with approximately 1 % prevalence among adults [55]. It is characterized by symmetrical synovitis, pannus formation, joint pain, stiffness, swelling, and destruction. The presence of RA is associated with an increased risk of the development of cardiovascular, neurological, and metabolic disorders [56]. Although, in recent years, the state of knowledge concerning the pathogenesis of RA has dramatically increased, the detailed mechanisms underlying this disease remain poorly understood, and therefore, the factors responsible for the onset and progression of this disorder are the subject of intense research. Undoubtedly, an important role is played by pro-inflammatory cytokines, particularly by TNFα, IL-1β, and IL-6 [55]. Taking into consideration the fact that articular and synovial AT is an inseparable component of human joints, local and systemic dysfunction in the synthesis, release, and receptor action of adipocyte-derived proteins was the aim of some recently published studies.

#### Leptin

It is assumed that Th1/Th2 imbalance plays an important role in the development of RA with a postulated detrimental role of an increased Th1 response in this disorder [57]. Leptin has been suggested to participate in modulating inflammation response through the induction of Th1 production of pro-inflammatory cytokines. Nevertheless, it is still poorly investigated whether leptin directly induces adipose tissue resident macrophages to release cytokines. On the other hand, pro-inflammatory cytokines raise circulating leptin levels, while in animal models leptin enhances the release of IL-6, IL-12, and TNFα from peritoneal macrophages [13], and TNFα from synovium, which suggests the existence of a local positive feedback between cytokines and leptin in joint tissues [58].

Most of the experimental studies conducted to date suggest a pro-inflammatory rather than protective action of leptin in joint inflammatory disorders (Table 1). Several studies confirm the protective impact of leptin deficiency on antigen-induced arthritis and a stimulatory effect of leptin on NO production [14, 58, 59]. Taking into consideration a well-documented degenerative effect of NO on joint cartilage, evoking the loss of chondrocyte phenotype, inducing chondrocyte apoptosis, and increasing activation of metalloproteases [60], an increase in leptin levels may deteriorate joint inflammation via the local generation of excessive amounts of NO. Surprisingly, the finding that the administration of exogenous leptin elevates IGF-1 and TGFβ secretion by rat knee joint cartilage may suggest that increased plasma leptin levels in obesity protect cartilage against degeneration [12]. Treatment with leptin in animal models of septic arthritis reduced the severity of joint damage, which may indicate that leptin in the synovial cavity exerts a protective effect against RA-induced joint destruction [61]. Regrettably, the results of the latter study were affected by a number of problems limiting their interpretation, that is, cross-sectional character of the study, which did not provide any information on the role of leptin in the course of the disease; assessment of the disease activity based only on plasma CRP levels; and lack of information about the body mass index of the subjects studied [62].

**Table 1:** Effect of adipokines on arthritis and non-arthritis joint tissues

Adipokine	Model	Results	References
Leptin	Leptin deficient ob/ob mice with antigen-induced arthritis	Less severe arthritis compared with control mice	Busso et al. [58]
		Reduction of T cell proliferation	
		Decrease in interferon-γ production	
		Lower levels of IL-1β and TNFα mRNA in the synovium of arthritis knees	
	ATDC5 mouse embryonic cells and human articular chondrocytes	Induction of NO-synthase expression and NO production in articular cartilage and synovium during treatment with leptin and interferon-γ	Otero et al. [14]
		Induction of NO production after leptin and IL-1 administration (mediated by PI-3 kinase, MEK-1, and p-38 kinase pathways)	Otero et al. [59]
Adiponectin	Rheumatoid synovial cells culture	Strong expression of adiponectin mRNA in synovial fibroblasts and articular adipose tissue	Ehling et al. [74]
		Induction of IL-8 expression	Kitahara et al. [76]
Resistin	NMRI mice with intra-articularly injected resistin	Development of arthritis with hypertrophy of the synovial layer and pannus formation	Bokarewa et al. [34]

*IL*-*1* interleukin, *NO* nitric oxide, *PI*-*3 kinase* phosphatidylinositide 3kinase, *MEK*-*1* mitogen-activated protein kinase 1

Despite generally consistent results of animal investigations, suggesting pro-inflammatory implications of leptin in the pathogenesis of RA, the data obtained from clinical studies are not so unambiguous. There are several studies that showed significantly elevated concentrations of leptin in patients with RA [61, 63–66]. Otero et al. [55] observed that plasma leptin levels increased markedly in patients with RA, independently of BMI value, while Targonska-Stepniak et al. [65] noted elevated leptin serum concentrations in patients with higher disease activity evaluated by DAS 28, ESR, and the number of tender joints. Also, Bokarewa et al. [66] reported elevated plasma leptin in RA, though no adjustment for BMI was made in this study. It was also noted that plasma concentrations of leptin were significantly higher than synovial fluid leptin, and this difference was particularly evident in non-erosive arthritis [66].

Although the majority of studies revealed high systemic and local leptin levels in patients and animals with RA, some other studies did not support these results. Anders et al. [67] found no differences between serum levels of this adipokine in RA and healthy subjects. A fasting-induced decrease in circulating leptin in RA patients was associated with CD4+ lymphocyte hyporeactivity and increased IL-4 serum concentration [68]. Reduced serum leptin levels in fasting RA patients resulted in a potentially beneficial shift toward Th2 cytokine production [7], increased insulin sensitivity, and rise in glucagon and glucocorticoid synthesis [69].

A study by Popa et al. [70] demonstrated the existence of an inverse correlation between severity of inflammation and circulating leptin levels in active RA, suggesting contribution of chronic inflammation to lowering plasma leptin concentration. Striking is the fact that this study did not reveal differences in leptinemia between the whole group of RA patients and healthy controls, which was explained by low inflammatory parameters at the time of inquiry.

According to the aforementioned investigations [67, 68], we may assume that improvements of symptoms may be related to a significant decrease in plasma leptin levels due to weight loss in the course of the disease. Nonetheless, it is not obvious whether the increase of plasma leptin in RA is just an effect of weight changes or it is rather a cause or a consequence of pathology in RA.

#### Adiponectin

The first report showing the existence of a correlation between adiponectin and RA was published in 2003 by Schaffler et al. [71]. The authors demonstrated that synovial fluid concentrations of adiponectin were significantly higher in patients with RA than in those with osteoarthritis (OA). In 2004, Berner et al. [72] evidenced that adiponectin is also expressed and secreted by osteoblasts, which corroborated previous opinions about the role of adiponectin in bone homeostasis. Elevation of synovial adiponectin in RA was later confirmed by other studies [72, 73]. Ehling et al. [74] demonstrated a strong expression of adiponectin mRNA in synovial fibroblasts and articular adipocytes of RA and OA patients. The same study showed that adiponectin induced, via p38 mitogen-activated protein kinase (MAPK) pathway, the synthesis of IL-6 and pro-matrix metalloproteinase-1 (pro-MMP-1). What is worth mentioning is that neutralization of TNFα activity by etanercept and adalimumab resulted in a marked reduction of IL-6 and pro-MMP-1. As the specific binding of entanercept and adalimumab to adiponectin was excluded, pro-inflammatory effects of adiponectin in the synovium were, at least in part, mediated by TNFα [74]. As TNFα was found to stimulate the p38 MAPK pathway, TNFα-directed therapy may modulate adiponectin action on the level of this signaling pathway [75].

Also, in other studies, adiponectin stimulated IL-6 production [29] and, in opposition to leptin and resistin, induced IL-8 expression [76] in rheumatoid synovial fibroblasts and chondrocytes [77] (Table 1). At the level of chondrocytes, adiponectin was found to exert pro-inflammatory effects by inducing the expression of inducible NO synthase and by stimulating the release of IL-6, MMP-3, MMP-9, and MCP-1 [78]. Another study reported that the mean levels of adiponectin and type 1 adiponectin receptor were higher in the synovial fluid of RA compared with OA patients. Interestingly, there were no statistically significant differences in serum adiponectin and the type 1 adiponectin receptor content between RA, OA, or healthy control subjects [79], while in endothelial cells adiponectin reduced the expression of TNFα-induced IL-8 [80]. Furthermore, in recent research by Kusunoki et al. [81], adiponectin enhanced production of prostaglandin E_2_ in synovial tissues obtained from patients with RA. All these results may suggest that adiponectin locally produced in joint tissues induces inflammation, which is consistent with previous opinions that adiponectin may exhibit some pro-inflammatory properties [82, 83]. This pro-inflammatory action of adiponectin may be limited to selected tissues, which would explain why, despite local changes in adiponectin levels, serum levels of adiponectin and adiponectin type 1 receptor did not differ between RA, OA, or healthy subjects. If this hypothesis is correct, plasma adiponectin levels may not reflect precisely the activity of this AT product in particular tissues. Alternatively, increased adiponectin production in autoimmune/chronic inflammatory conditions might be secondary to inflammation-induced catabolic responses occurring in RA, which are absent in inflammation associated with obesity [24, 84].

There are some reports indicating that locally abnormal activity of adiponectin in joint tissues is not only associated with the presence, but also determines the severity of RA. Recent research by Ebina et al. [85] has shown that serum adiponectin levels were higher in patients with severe RA than in mild RA and control groups (RA was graded on the basis of the extent of joint destruction). It should be underlined that the difference in adiponectin levels between subjects with severe and mild RA did exist, despite higher CRP levels and the use of a higher dose of oral prednisolone by the patients with mild RA [85] (both CRP and corticosteroids have been reported to markedly inhibit adiponectin [86]). Similarly, there was a strong positive correlation between serum adiponectin levels and progression of radiographic joint destruction, including enhanced radiographic erosions, and joint space narrowing [87, 88] As a recent study by Klein-Wieringa et al. [89] shows, baseline serum levels of adiponectin can predict radiographic progression independently of the presence of anti-cyclic citrullinated peptide antibodies and BMI. These findings suggest that circulating adiponectin and/or adiponectin produced locally by intra-articular adipocytes may play a role in the degradation of extracellular matrix components. These local pro-inflammatory and erosive effects of adiponectin may result from the stimulation of the NF-κB pathway [29] and/or osteoclastogenesis [90], respectively. On the other hand, as suggested by Fantuzzi [24], the catabolic state accompanied by joint destruction, especially in large joints, may be a significant determinant of hyperadiponectinemia.

The results presented above suggest that adiponectin may be a target for the treatment of RA. However, adiponectin would not be called “a controversial hormone” if there were no contradictory opinions about its function. Despite many data suggesting the pro-inflammatory action of adiponectin in joints [29, 74, 78], it cannot be completely excluded that high local and systemic levels of adiponectin help suppress inflammation in patients with RA. In accordance with this hypothesis, in collagen-induced arthritis mice and RA synovial fibroblasts, intra-articularly injected adiponectin significantly mitigated the severity of the arthritis and histopathological findings indicative of RA [91].

Unfortunately, one of the serious limitations of the studies conducted to date is that they measured almost exclusively total adiponectin. The ambiguous impact of this adipokine on arthritis [29, 74, 78, 91] may, in part, be explained by different biologic functions of various adiponectin isoforms. The latest findings by Chedid et al. [92] are in line with this assumption. The authors have demonstrated that adiponectin and its globular fragment differentially modulated the oxidative burst of primary human phagocytes. Contrary to full-length adiponectin, its globular form, constituting about 25 % of adiponectin in synovial fluid, enhanced reactive oxygen species production and phagocytic NADPH oxidase-2 expression in the plasma membrane, with a concomitant increase in p47(phox) phosphorylation. Interestingly, the same study has shown that LMW adiponectin was more abundant in synovial fluid than in serum from RA patients [92], and these findings suggest that joint inflammation in RA may be associated with an imbalance between different isoforms of adiponectin.

Although at present, the number of premises indicating pro-inflammatory function of adiponectin in RA patients seems to prevail over data showing its protective action, the association between adiponectin and RA is far from being completely understood. Because no firm conclusions can be drawn; more research in this field is undoubtedly required, particularly with reference to the role of adiponectin isoforms.

#### Resistin

Although most reports concerning resistin focused on its function in the metabolic syndrome, obesity, and IR, there is some evidence on its role in RA and other inflammatory diseases. The pioneering work by Schaffler et al. [71] from 2003 showed that not only adiponectin is elevated in the synovial fluid of RA patients, but also resistin levels in the synovium are about 10 times higher than in OA subjects. Resistin, as it was shown by Bokarewa et al. [34], accumulates locally in the inflamed joints of RA patients. Furthermore, the hypothesis of pro-inflammatory resistin function was confirmed by the development of arthritis after resistin injection into the joints of healthy mice [34] (Table 1). Interestingly, plasma resistin concentrations remained low, suggesting the local intra-articular action of this agent. Although, there are reports showing no difference in serum resistin levels between RA patients and healthy subjects [64], two successive studies reported a positive correlation between circulating resistin levels and the severity of inflammation in RA [93, 94]. Furthermore, Migita et al. [93] observed correlations between serum resistin and CRP, ESR and TNFα in patients with RA, which is consistent with earlier findings by Schaffler et al. [71] and a recent report by Forsblad d’Elia et al. [94]. This study also revealed a strong positive correlation between resistin and IL-1 receptor antagonist, the serum level of which is elevated in many rheumatic diseases [95], whereas bone mineral density was inversely correlated with serum resistin. The fact that in humans resistin levels positively correlated with coronary atherosclerosis occurrence may suggest a role of resistin in the inflammation-based etiology of atherosclerosis in RA [94]. Various resistin levels in serum and synovial fluid of RA patients [34, 64, 71, 93, 94] may be due to or may contribute to differences in RA disease activity.

#### Other adipokines

The role of the remaining adipokines in the development and progression of RA is even less understood than the role of adiponectin, leptin, and resistin.

Plasma levels of visfatin were found increased in patients with RA [64]. Visfatin was evidenced to induce chemotaxis and the production of IL-1, TNFα, IL-6, together with costimulatory molecules by CD14C monocytes, and to increase monocyte ability to induce alloproliferative responses in lymphocytes [96, 97]. These features may suggest that increased visfatin production contributes to the pathogenesis of RA. Because visfatin is suggested to be a part of a compensatory mechanism facilitating lipid accumulation in intra-abdominal depots, it may protect the patient against the development of rheumatoid cachexia [64].

In a study by Senolt et al. [48], synovial fluid vaspin levels were higher, while omentin levels were lower in RA patients than in OA patients. Synovial fluid vaspin tended to correlate with the activity of RA assessed by DAS28, but not with serum CRP or a number of leukocytes in synovial fluid. On the other hand, synovial fluid levels of omentin correlated with serum anti-citrullinated peptide antibodies and with IgM-rheumatoid factor [48]. Elevated serum vaspin levels in RA patients have also been recently demonstrated by Ozgen et al. [98].

In light of latest research, it seems that novel adipokines, such as chemerin, LCN 2, and SAA3, may also play some role in the development and progression of rheumatic diseases. These adipose tissue proteins are partially produced by murine and human chondrocytes [53] and their production is up-regulated by pro-inflammatory cytokines and lipopolysaccharide [52].

Chemerin stimulates leukocyte migration to sites of inflammation. It induces the release of C–C chemokine ligand 2 and enhances the expression of toll-like receptor 4, which is a well-known inhibitor of cartilage biosynthetic activity [52]. A recent study by Eisinger et al. [99] showed that chemerin is present in synovial fluids of RA, OA, and psoriatic arthritis patients. Although the significance of chemerin in innate immune system–associated joint inflammation seems probable, its role in the pathogenesis of rheumatic diseases is still unknown.

LCN 2 forms molecular complexes with MMP-9 and, by protecting this MMP from autodegradation [100], may contribute to the degeneration of cartilage. LCN 2 is highly susceptible to upregulation by IL-1β and, therefore, arouses interest as a potential biomarker of cartilage degeneration in arthritic diseases.

SAA3 increases MMP-1 secretion in rabbit fibroblasts, while the human analogue of SAA3 (A-SSA) can induce MMP-1 and MMP-13 in human chondrocytes [101]. Significant is the finding that high concentrations of A-SSA have been detected in the inflamed synovium of RA and OA patients, which suggests that SAA3 is involved in cartilage degeneration in rheumatic diseases [101].

### Systemic lupus erythematosus

SLE is a chronic, multi-system, inflammatory connective tissue disorder with no single diagnostic marker and relatively poorly understood pathogenesis. It is also an autoimmune disease characterized by the production of auto-reactive antibodies to various components of the cell nucleus. It is identified on the basis of a combination of clinical and laboratory criteria, where inflammation, vasculitis, immune complex deposition, and vasculopathy are main pathological findings [102, 103]. It is noteworthy that inflammatory processes in SLE involve not only vessel wall, skin, and serous membranes, but also joints, kidneys, liver, blood morphological elements, and various neuropsychiatric presentations [102].

Patients with SLE often fulfill the criteria of the metabolic syndrome, and have accelerated atherosclerosis development and higher prevalence of IR [104]. Taking into consideration the significance of adipokines in inducing these disorders, it seems that AT products may exhibit a significant impact on the development and progression of SLE. Interestingly, this disease affects mainly women. It is in line with higher circulating levels of leptin in this gender, which is caused by differences in body composition and hormone distribution between sexes [105]. Although the number of studies assessing the role of adipokines in SLE is much more limited than in RA, they have shown some relationships between adipokines and inflammatory joint diseases.

#### Leptin

The results of studies assessing plasma leptin concentration of patients with SLE are generally consistent and indicate that high serum leptin levels might contribute to systemic inflammation in SLE patients [62, 106, 107]. It has been shown that serum leptin was higher in women with SLE than in healthy controls [106], even after adjustment for hypertension, hyperlipidemia, and diabetes [108]. Chung et al. [109] also showed that high serum leptin levels correlated with IR, the presence of the metabolic syndrome, and high levels of CRP, ESR, LDL cholesterol, and triglycerides. Interestingly, the authors emphasized that the difference in serum leptin levels between patients with SLE and controls was independent of age, race, sex, and BMI. These findings suggest that leptin production in SLE is influenced by additional factors, which may be causatively linked to the initiation and progression of this disorder [109]. Despite higher serum concentrations in SLE patients, the association between leptin levels and disease activity (*stratified according to the MEX*-*SLEDAI scores*) has not been evidenced [110]. Unexpectedly, no differences in leptin levels between patients with SLE and healthy subjects were found in the research by Wislowska et al. [111], who also observed lower serum leptin levels in SLE patients with neurological disorders and arthritis than in individuals free from them.

Interestingly, Härle et al. [112] found the presence of an inverse correlation between serum levels of leptin and androstenedione in both SLE and RA patients. This finding, suggesting that leptin inhibits androstenedione secretion, may partially explain hypoandrogenism, which is frequently observed in patients with chronic inflammatory diseases [112].

#### Adiponectin

It has been demonstrated that the risk of IR in patients with SLE is higher compared with age-matched healthy controls [113]. Regarding the fact that adiponectin enhances insulin sensitivity, this adipokine may be involved in the pathogenesis of SLE and the development of its complications. In a report by Sada et al. [108], serum levels of adiponectin were significantly elevated in SLE without IR compared with healthy controls, and lower levels of adiponectin were noted in SLE patients with IR compared to SLE subjects without IR. Although adiponectin was negatively correlated with IR in SLE subjects, serum levels of this adipokine in SLE patients with reduced insulin sensitivity exceeded those observed in healthy subjects. Though the authors were reluctant to draw any definite conclusion, they speculated that the stimulating effect of adiponectin on insulin sensitivity might be impaired in some patients with SLE. Increased plasma concentrations of adiponectin in patients with SLE were also observed by Chung et al. [109]. Interestingly, despite this fact, these authors found the negative correlation between plasma adiponectin and BMI, presence of metabolic syndrome, systolic blood pressure, and dyslipidemia in patients suffering from this disorder. In the study by Rovin et al. [110], the authors showed that serum adiponectin levels are higher in patients with renal SLE than in healthy controls and in patients with non-renal SLE. Furthermore, during renal SLE flare, urine adiponectin loss was markedly increased. Noteworthy is the fact that HMW adiponectin isoform has recently been found in urine of patients with active lupus nephritis, but not in urine of healthy individuals, and its content correlated with lupus severity [30]. Because HMW isoform has been demonstrated to induce IL-8 and MCP-1 production, higher amounts of this isoform may result in pro-inflammatory properties of adiponectin in this group of patients.

#### Resistin

There is no agreement as for concentrations and function of resistin in SLE, because of a limited number of studies and their inconsistent results. Only in one report, the authors found the existence of a relationship between serum resistin levels and the severity of inflammation, bone mass density (BMD), and renal function in SLE patients [114]. Serum resistin levels correlated also with low HDL cholesterol and high IgG levels, as well as with elevated levels of pro-inflammatory cytokines—IL-1β, IL-6, and TNFα and soluble IL-6 receptor in serum. The association between resistin, ESR, and complement component 3 (C3) levels, observed in this study, may reflect disease activity [114]. In other studies, correlation between SLE occurrence and serum resistin concentrations was not found [109, 115, 116]. However, in one of these studies [109], a weak association between resistin and ESR was revealed. Due to a small number of studies and their limitations, at present it is too vague to make any firm conclusions concerning the involvement of resistin in SLE.

### Other connective tissue disorders

#### Sjögren’s syndrome

Sjögren’s syndrome (SS) is an autoimmune disorder which is characterized by chronic dysfunction and destruction of salivary and lacrimal glands, leading to persistent dryness of the mucosa [117]. Destruction of the salivary glands is accompanied by the development of adipose tissue and fibrotic tissue, which may suggest the involvement of adipocytes in the pathogenesis of this disorder. There are only a few studies investigating adipokine levels/production in patients with SS. Toussirot et al. [118] observed a marked increase in circulating adiponectin concentration in a small group of patients with SS. Katsiougiannis et al. [119] investigated the expression of adiponectin in minor salivary gland biopsy specimens obtained from patients with SS and controls. The authors observed increased constitutive secretion of adiponectin by salivary gland epithelial cells from patients with SS compared with controls, but did not find any changes in the production of adiponectin by adipocytes. Another study [120] revealed elevated saliva resitin levels, despite unaltered plasma levels of this adipokine, as compared with healthy controls, which corresponded to the intensity of lymphocytic inflammation in salivary glands [120]. It seems that locally produced adiponectin exerts anti-proliferative and anti-apoptotic effects on salivary gland epithelial cells through the activation of AMP-activated protein kinase [121]. These findings indicate that adiponectin and resistin may locally regulate immune processes, regardless of their metabolic functions. They also suggest that plasma adipokine concentrations seem not to be as sensitive indicators of local inflammatory processes as their saliva levels.

#### Ankylosing spondylitis

Ankylosing spondylitis (AS) is a chronic inflammatory disease with 0.2–0.9 % prevalence among the population [122]. It mainly affects the axial skeleton, but it is also a risk factor for cardiovascular disease. Serum concentration of adiponectin has been found to be unaffected in AS patients compared to controls [123]. Some [122, 123], but not other, [124] authors showed a significant decrease in serum leptin concentration in patients with AS and a reduction in adipose tissue mass [122]. Sari et al. [122] even speculated that decreased leptin levels might be used as a marker of disease activity in AS. However, taking into account the limited number of studies investigating serum levels of leptin, adiponectin, and other adipokines in AS and their inconsistent results, as well as the fact that no study has assessed synovial fluid adipokine concentration in this disorder, the role of adipose tissue hormones in the development and progression of AS requires better understanding.

#### Systemic sclerosis

Very little is known about relevance of adipokines in the pathogenesis of systemic sclerosis. Individuals suffering from this disorder had lower serum leptin levels compared with healthy subjects [125]. However, taking into consideration the fact that the participants of this study tended to have decreased body mass, while leptin levels correlated with BMI, low leptin levels may have reflected body weight loss rather than inflammatory process directly responsible for the development of this disorder.

Serum adiponectin levels and adiponectin mRNA levels in skin tissues were reduced in patients with diffuse cutaneus scleroderma, having higher total skin thickness score and higher incidence of pulmonary fibrosis. Although these findings suggest that serum adiponectin levels may be a useful biomarker for fibrotic condition in systemic sclerosis, further studies are required to confirm this hypothesis [126].

### Adipokines and treatment of connective tissue diseases

The issue of whether anti-TNFα therapy affects leptin, adiponectin, and resistin concentrations in patients with RA have been investigated in several recent studies [127–132]. Because the production of some adipokines is regulated by TNFα, IL-1, and other pro-inflammatory cytokines, biological treatment of RA should at first glance change adipokine production and release. Unfortunately, in the case of leptin, all conducted studies showed that anti-TNFα therapy has either limited impact [127] or even does not [128, 129] change its plasma concentration. Futhermore, Gonzalez-Gay et al. [130] showed that serum leptin levels in infliximab-treated RA patients did not correlate with the parameters determining disease activity and the severity of inflammation. Although in the said study circulating leptin levels during anti-TNFα therapy correlated with the content of AT, a similar correlation was observed also in non-RA control subjects. Interestingly, in an earlier study of the same research group, the authors observed a rapid reduction of serum resistin levels in RA patients treated with anti-TNFα therapy, which may support a potential role of this adipokine in the inflammatory cascade in RA [131]. Studies assessing adiponectin levels during anti-TNFα therapy in RA patients provided contrasting results. Härleet al. [129] did not find any changes in serum adiponectin levels during anti-TNFα therapy with adalimumab, while Nagashima et al. [132] observed increased plasma levels of this protein after treatment with etanercept and infliximab. The latter results may be explained by a direct effect of TNFα blockade, because both TNFα and adiponectin reciprocally inhibit the production of each other [132]. As high TNFα and resistin as well as low adiponectin levels are associated with increased cardiovascular risk [133], pharmacological blockage of TNFα, apart from exhibiting anti-inflammatory effect, may lead to a reduction in cardiovascular morbidity and mortality. Unfortunately, owing to a very limited number of studies assessing the relationship between anti-cytokine agents and adipokine production, this hypothesis is a bit speculative and needs to be verified in future studies.

### Therapeutic perspectives

Since their discovery, most researchers have paid attention to therapeutic application of adipokines, their analogs and derivatives in the treatment of metabolic disorders. The results of many studies described in this review indicate that the modification of the adipokine network should be considered as one of future treatment options for connective tissue diseases.

The involvement of leptin in the pathogenesis of inflammatory and autoimmune diseases allows us to believe that prevention of leptin-induced inflammation may bring benefits to both RA and SLE subjects. This may be obtained using high-affinity leptin-binding molecules (analogously to the soluble TNFα receptors used to treat RA) or by blocking the leptin receptor with monoclonal humanized antibodies or mutant leptins, which are able to bind to this receptor without activating it [7]. Another challenging possibility of leptin modulation is the use of epigenetic therapy or targeted gene therapy. In line with this possibility, epigenetic therapy produced an impact on leptin expression in normal chondrocytes, while targeted gene therapy using small interference RNA transferred with liposomes dramatically inhibited MMP‐13 expression in osteoarthritic chondrocytes [134]. Whichever strategy is chosen, it should not modify the influence of leptin on food intake in order to avoid the development of hyperphagia and obesity. Because of unresolved doubts as to the role in the regulation of inflammatory processes, more controversial remains the question of the use of drugs affecting the adiponectin system. Moreover, owing to the existence of various forms of adiponectin, their actions may differ markedly, and therefore, using adiponectin-directed agents in RA or SLE patients requires further study.

A more promising target for the development of novel therapeutics for the treatment of inflammatory diseases seems to be visfatin. Neutralization of visfatin by its inhibitor (APO866) effectively reduced arthritis severity in mice with comparable activity to etanercept, and decreased pro-inflammatory cytokine (IL-1β and IL-6) secretion in affected joints [135]. Another interesting direction for a new treatment option for connective tissue disease is inhibiting the synthesis, release, and action of resistin by anti-resistin agents.

It should be remembered that the mechanism of action of each adipokine seems to consist not only of its direct effect, but also of its indirect effect resulting in the modulation of other adipose tissue protein systems. The presence of numerous adipokine systems and the possibility of numerous interactions between them result in precise response to energetic and immunological balance changes and consequently in the precise regulation of organism homeostasis. This fact, although favorable in normal conditions, may hamper the efficacy of the treatment of immunological disorders because the pharmacological manipulation of the activity of a single system alters the activity of other systems, thus neutralizing the therapeutic effect achieved. This may justify the use of agents affecting more than one adipokine system in the treatment of connective tissue disorders.

## Discussion

Most of the data presented by different research groups showed increased levels of leptin, adiponectin, and resistin in plasma and synovial fluid in RA and SLE (Table 2). Nevertheless, despite efforts, it still remains obscure whether the observed disturbances in various adipokine systems in subjects with connective tissue diseases contribute to their development or only mirror the presence or activity of inflammatory process, which itself is induced by other pro-inflammatory factors. It remains unanswered why some adipokines, particularly adiponectin, produce pro- or anti-inflammatory effects in connective tissue diseases and why clinical studies assessing plasma and synovial fluid concentrations of various adipokines provided contrasting results. Plasma levels of adipokines might tell us too little about their role in connective tissue disorders due to the fact that adipokine secretion depends on numerous factors, such as different anatomic distribution of adipose tissue, adipocyte size, and hormonal regulation. Moreover, different forms of the same protein might produce different effects on inflammation. Adipokine effects on synovial tissues might differ from their known metabolic or cardiovascular effects, which implies that some re-appraisal of adipokine role may need to take place. Because data regarding seemingly well-known adipokines tend to be ambiguous, it appears that a better understanding of the role of “new adipokines” (visfatin, acylation-stimulating protein, vaspin, apelin, fasting-induced adipose factor, retinol-binding protein-4, chemerin, lipocalin 2, and serum amyloid A3) and their cooperation with “old adipokines” could throw light on the pathogenesis of inflammatory processes.

**Table 2:** Adipokine concentration in rheumatoid arthritis and systemic lupus erythematosus

	Plasma concentration^a^	Synovial fluid concentration^a^	Comments	References
*Rheumatoid arthritis *(*RA*)				
Leptin	↑	↑	ESR higher in moderate disease activity RA group compared to low activity group (*P* < 0.001)	Seven et al. [63]
	↑	–		Otero et al. [64]
	↑	–		Targonska-Stepniak et al. [65]
	↑	–	Disease activity evaluated by DAS 28, ESR, and the number of tender joints	Bokarewa et al. [66]
	0	–	No adjustment for BMI	Anders et al. [67]
	0	–		Popa et al. [70]
Adiponectin	–	↑	Stimulation of IL-6 and pro-MMP-1 production in synovial fibroblasts	Tang et al. [29]
	0	↑	Compared to OA patients	Schaffler et al. [71]
	↑	↑	Compared to OA patients	Senolt et al. [73]
	0	↑	Compared to OA patients	Tan et al. [79]
	↑	–	Serum adiponectin levels were higher in patients with severe RA than in mild RA and control groups	Ebina et al. [85]
Resistin	0	↑	Positive correlation between synovial resistin levels and severity of inflammation defined by intra-articular white blood cell count and IL-6 levels	Bokarewa et al. [34]
	↑	↑	Positive correlation between serum resistin and: (1) CRP, (2) DAS 28	Senolt et al. [136]
	–	↑	Compared to OA patients Positive correlations between synovial resistin levels and (1) ESR, (2) CRP	Schaffler et al. [71]
	↑	–	Positive correlations between serum resistin and (1) CRP, (2) ESR, (3) TNFα	Migita et al. [93]
	0	–	Positive correlation between resistin and IL-1Ra	Forsblad d’Elia et al. [94]
*Systemic lupis erythematosus *(*SLE*)				
Leptin	↑	–		Garcia-Gonzalez et al. [106]
	↑	–		Sada et al. [108]
	↑	–	Positive correlation between high serum leptin levels and (1) IR, (2) the presence of the metabolic syndrome, (3) CRP, (4) ESR, (5) LDL cholesterol, (6) triglycerides	Chung et al. [109]
	↓	–	Signifcantly lower serum leptin levels in SLE patients with arthritis and central nervous system (CNS) involvement in comparison with SLE patients without arthritis and CNS involvement	Wislowska et al. [111]
Adiponectin	↑	–	Lower levels of adiponectin SLE patients with IR compared to SLE subjects without IR	Sada et al. [108]
	↑	–	Negative correlation between plasma adiponectin and (1) BMI, (2) presence of metabolic syndrome, (3) systolic blood pressure, (4) dyslipidemia	Chung et al. [109]
	↑	–	Serum adiponectin levels higher in patients with renal SLE than in healthy controls and in patients with non-renal SLE	Rovin et al. [110]
Resistin	0	–	Weak positive correlation between resistin and ESR	Chung et al. [109]
	0	–	Positive correlation between resistin and (1) creatinine, (2) IgG, (3) IL-1β, IL-6, and TNFα and soluble IL-6 receptor in serum Negative correlation between resistin and (1) GFR, (2) HDL cholesterol, (3) BMD, (4) complement levels	Almehed et al. [114]
	0	–		Vadacca et al. [115]
	0	–		De Sanctis et al. [116]

“↑”—elevated concentration of adipokine, “↓”—decreased concentration of adipokine, “0”—no differences in concentration between patients and controls, “–”—data not assessed, *OA* osteoarthritis, *ESR* erythrocyte sedimentation rate, *CRP* C-reactive protein, *DAS*-*28* disease activity score, *TNF*α tumor necrosis factor α, *IR* insulin resistance, *GFR* glomerular filtration rate, *BMI* body mass index, *BMD* bone mass density, *pro*-*MMP*-*1* pro-matrix metalloproteinase-1, *IL*-*6* interleukin 6, *IL*-*1Ra* interleukin 1 receptor antagonist

^a^Patients versus healthy controls unless other control group is stated in comments

Establishing the exact position of adipose tissue proteins in the pathogenesis of connective tissue diseases, apart from pathophysiological implications, arouse deep interest because adipokine systems may in the future become potential targets for new effective and safe pharmacological agents for the treatment of RA, SLE, and other autoimmune disorders. New developments concerning AT hormonal function has not provided definite answers to numerous questions and paradoxically even raised additional queries. Therefore, further studies are required to establish the proper role of adipokines in connective tissue diseases.
